# Closed Genome Sequence Obtained Using Hybrid Nanopore/Illumina Assembly of a Bacillus anthracis Isolate from an Animal-Skin-Drum-Associated Anthrax Case in the United Kingdom

**DOI:** 10.1128/MRA.00802-18

**Published:** 2018-10-04

**Authors:** Steven T. Pullan, Rory W. Miles, Kuiama Lewandowski, Richard Vipond

**Affiliations:** a Public Health England, National Infection Service, Salisbury, Wiltshire, United Kingdom; Loyola University Chicago

## Abstract

Hybrid *de novo* assembly of Illumina/Nanopore reads produced a complete closed genome sequence of the chromosome and two virulence plasmids of a Bacillus anthracis isolate from a fatal anthrax case in the United Kingdom linked to imported animal skins/drums; this provides a high-quality representative sequence for this lineage.

## ANNOUNCEMENT

Bacillus anthracis is the causative agent of the zoonotic disease anthrax. Despite its relatively rare occurrence in humans, it has gained notoriety due to its potential use as a bioweapon (1). In the United Kingdom, the most recent human cases have been associated with the intravenous use of contaminated heroin (2, 3) or the importation of animal hides for drum making (4). We previously characterized multiple isolates from two animal-skin-drum-associated anthrax cases in the United Kingdom, utilizing Illumina sequencing and a reference mapping-based approach to identify core genome single-nucleotide polymorphisms (SNPs). Phylogenetic analysis showed that these strains (along with another collected from a similar U.S. case [5]) formed a novel branch on the global B. anthracis phylogeny that was distinct from previously characterized West African strains, despite this being the likely origin of the contaminated animal skins (6). In order to produce a more complete reference sequence for this novel lineage, we used the Oxford Nanopore MinION genomic DNA sequencing kit (SQK-MAP006) on a MinION Mk1 device, with 1 μg of DNA (isolated as described previously) as input to produce 11,231 2D reads with a mean length of 7.5 kb; the predicted genome coverage depth was ∼16×. Fasta sequences were extracted from FAST5 files using poRe (7) and used along with the existing Illumina data (∼50× read depth [6]) in a hybrid assembly using Unicycler (8). Assembly of the Illumina data alone using SPAdes (9) produced an assembly of 87 scaffolds, including a complete pX02 plasmid sequence but multicontig chromosomal and pX01 sequences (comprised of 82 and 4 contigs, respectively). The addition of the Nanopore data and use of the Unicycler assembly pipeline produced complete circular sequences for the chromosome (5,288,599 bp) and the pX01 and pX02 plasmids (180,666 bp and 94,967 bp, respectively).

### Data availability.

These data are available under NCBI BioProject number PRJNA287512, BioSample number SAMN03790807, and GenBank accession numbers CP029805 (London_499 chromosome), CP029806 (London_499 pX01), and CP029807 (London_499 pX02).
